# Gut Microbiota: Association with NAFLD and Metabolic Disturbances

**DOI:** 10.1155/2015/979515

**Published:** 2015-05-19

**Authors:** E. Lau, D. Carvalho, P. Freitas

**Affiliations:** Department of Endocrinology, Diabetes and Metabolism, Centro Hospitalar São João, Institute for Research and Innovation in Health Sciences, Faculty of Medicine, University of Porto, Alameda Professor Hernâni Monteiro, 4200-319 Porto, Portugal

## Abstract

Nonalcoholic fatty liver disease is the hepatic expression of metabolic syndrome, being frequently associated with obesity, insulin resistance, and dyslipidemia. Recent lines of evidence have demonstrated a role of gut microbiota in insulin resistance, obesity, and associated metabolic disturbances, raising the interest in its relationship with NAFLD pathogenesis. Therefore, intestinal microbiota has emerged as a potential factor involved in NAFLD, through different pathways, including its influence in energy storage, lipid and choline metabolism, ethanol production, immune balance, and inflammation. The main objective of this review is to address the pathogenic association of gut microbiota to NAFLD. This comprehension may allow the development of integrated strategies to modulate intestinal microbiota in order to treat NAFLD.

## 1. Introduction

Nonalcoholic fatty liver disease (NAFLD) is a very common disease, ranging from simple hepatic steatosis, characterized by excessive fat deposition in hepatocytes without any inflammation or necrosis to nonalcoholic steatohepatitis (NASH), characterized by steatosis and hepatic inflammation [1]. NAFLD is the hepatic expression of metabolic syndrome, being frequently associated with obesity, insulin resistance, and dyslipidemia [2]. Thus, its prevalence rises in parallel with the worldwide metabolic diseases epidemic, frequently developing on the background of obesity [3].

Although the pathogenesis of NAFLD is not completely understood, considerable progress has been made in recent years in elucidating the mechanisms responsible for liver injury. Initial theories were based on a “2-hit hypothesis” [4]. The “first hit” was characterized by hepatic triglyceride accumulation, which increases susceptibility of the liver to injury mediated by “second hits,” such as inflammatory cytokines/adipokines, mitochondrial dysfunction, and oxidative stress, which in turn lead to steatohepatitis and/or fibrosis [5].

**Figure 1:**
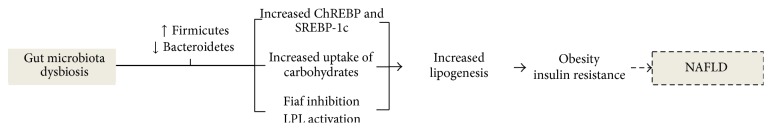
Schematic view of how the gut microbiota affects host fat storage and insulin resistance, which may result in NAFLD. The microbiota acts through an increase in the transactivation of lipogenic enzymes by liver carbohydrate response element binding protein (ChREBP) and sterol regulatory element binding protein 1 (SREBP-1), an increase in the uptake of dietary polysaccharides and through Fiaf inhibition with increased LPL activity in adipocytes, thereby promoting increase of hepatic lipogenesis and storage of calories harvested from the diet into fat.

The gastrointestinal tract harbors the largest number of bacteria, representing more than 150-fold their eukaryotic nuclear genome [6]. This “microbial organ” is recognized to perform a variety of physiological functions, from protective functions to metabolic regulation, including an active part on glucose and lipid metabolism [7]. Recent lines of evidence suggest a role of gut microbiota in insulin resistance and obesity [8–11], raising the interest of gut microbiota as an active intervenient on NAFLD (Figure 1). Microbiota seems to induce obesity through several mechanisms: ability of microbial products such as acetate and propionate to signal via intestinal epithelial receptors; increased intestinal permeability with translocation of bacterial products resulting in high level of metabolic inflammation; and caloric salvage by some microbes being able to extract calories from food [12]. There is a close anatomical and functional relationship between gut and liver, through portal circulation, favoring bidirectional influences [13]. Liver receives approximately 70% of its blood supply from the intestine, representing the first line of defense against gut-derived antigens [13]. Thus, gut microbiome may play an important role in the maintenance of gut-liver axis health and in NAFLD pathogenesis.

In the background of obesity and insulin resistance, this systematic review aims to explore the relationship which links microbiota to NAFLD.

## 2. Methods

**Figure 2:**
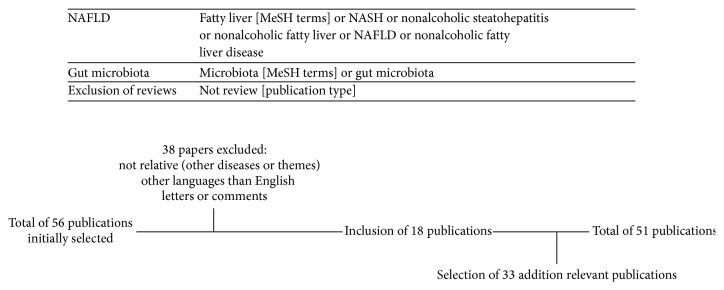
Search strategy in PubMed and studies selection.

A literature search was conducted with the aim of finding original experimental, epidemiological, and clinical studies on the association between gut microbiota and NAFLD. The search strategy used in PubMed, including the studies selection, is shown in Figure 2. Additional papers were identified in the reference lists of selected articles that met the inclusion criteria. Inclusion criteria were as follows: clinical studies with participants of any sex or ethnic origin with NAFLD/NASH diagnosed on the basis of radiological/histological evidence of fatty liver and epidemiological or experimental studies, regarding association between NAFLD/NASH and gut microbiota. Exclusion criteria were as follows: other causes of hepatic steatosis, such as alcoholic hepatic steatosis or viral hepatitis, and papers written in other languages than English.

All articles were read in full. Two independent investigators assessed papers for inclusion. Disagreement was resolved by discussion.

## 3. Results

Experimental and clinical studies have explored the pathogenic association between gut microbiota and NAFLD. A summary of studies, both in animal models and humans, are resumed in Tables 1 and 2, respectively.

### 3.1. Gut Microbiota Profile and Driven Mechanisms Associated with NAFLD

#### 3.1.1. Experimental Data

Experimental data have addressed the role of gut microbiota in the regulation of immune balance, low-grade inflammation, gut permeability, and lipid metabolism on NAFLD.

Due to its anatomical links to the gut, the liver is constantly exposed to gut-derived bacterial products. Immune cells like Kupffer cells recognize molecular pathogen-associated molecular patterns (PAMPs) through pattern recognition receptors, for example, toll-like receptors (TLR), thereby playing an important role in the protection against systemic bacterial [32].

Dietary fructose intake is associated with NAFLD development [33]. In a fructose-induced NAFLD mice model, hepatic steatosis was associated with a significant induction of TLR 1–4 and 6–8 [31]. Fructose-fed animals also had significantly higher number of F4/80 positive cells, a macrophages marker, and lower protein concentration of occludin, a tight junction protein [31]. Furthermore, the activation of TLRs is associated with an increase in levels of endotoxemia, produced by Gram-negative bacteria and lipopolysaccharides (LPS), emphasizing their role in intestinal permeability regulation and bacterial translocation [20]. Basically, LPS and other microbial components, in the intestine, bind to the specific receptor-activating TLRs signaling, triggering the activation of pro-IL-1*β* and pro-IL-18, which are processed into their active forms, and subsequently induce inflammation and fibrosis [20]. Taken together, these data support the notion that the onset of fructose-induced-NAFLD may be linked to an increase in intestinal translocation of microbial components, related to increased intestinal permeability and also dependent on an inflammatory response, through the increase of F4/80 positive cells and induction of several TLRs [20, 31, 33].

Inflammasomes are cytoplasmic multiprotein complexes consisting of caspase 1, PYCARD, NALP, and sometimes caspase 5, which act like sensors of endogenous or exogenous PAMPs or damage-associated molecular patterns (DAMPs). They regulate the activation of effector proinflammatory cytokines, such as pro-IL-1*β* and pro-IL-18, and are expressed in myeloid cells and are a component of the innate immune system [21]. The exact composition of an inflammasome depends on the activator, which initiates inflammasome assembly; for example, dsRNA will trigger one inflammasome composition whereas asbestos will assemble a different variant. The inflammasome promotes the maturation of the inflammatory cytokines Interleukin 1*β* (IL-1*β*) and Interleukin 18 (IL-18). Thus, the inflammasome is responsible for activation of inflammatory processes and has been shown to induce cell pyroptosis, a process of programmed cell death distinct from the immunologically silent death mechanism that characterizes apoptosis. Pyroptosis is an intriguing inflammasome-mediated host defense mechanism, which prevents intracellular replication of pathogens, by releasing their intracellular content into circulation and therefore targeting the destruction of surviving bacteria by phagocytes and neutrophils. Different animal models reveal that inflammasome deficiency-associated changes in the gut microbiota composition were associated with exacerbated hepatic steatosis and inflammation through the influx of TLR4 and TLR9 agonists into the portal circulation. Subsequently, hepatic TNF-*α* expression was enhanced, inducing NASH progression [21]. Porphyromonadaceae was found to be increased in inflammasome-deficient mice and associated with exacerbated hepatic steatosis and inflammation [21]. le Roy et al. had also showed higher concentrations of Porphyromonadaceae in a mouse model of hepatic steatosis [22].

The microbiota also regulates energy and lipid metabolism, directing the host to a rapid increase in body fat content, despite reduced chow consumption, and to increase hepatic production of triglycerides [11]. Conventionalization of germ-free mice promoted absorption of monosaccharaides and short chain fatty acids by fermentation and thus increased* de novo* hepatic lipogenesis and fat storage, by increasing liver carbohydrate response element binding protein (ChREBP) mRNA and regulating lipoprotein lipase activity [11]. Additionally, it seems that saturated fat stimulates hepatic steatosis and affects gut microbiota composition by an enhanced overflow of dietary fat to the distal intestine [23]. A saturated fat diet based on palm oil increased liver fat accumulation, reduced microbial diversity, and increased the Firmicutes-to-Bacteroidetes ratio [23]. A diet-induced elevation of lipid metabolism-related genes in the distal small intestine was also observed confirming the overflow of palm oil to the distal intestine [23]. Interestingly, a recent study has demonstrated that gut microbiota markedly impacts the lipid metabolism in the liver, independently of obesity [22].

The steatosis that first characterizes NAFLD may progress toward steatohepatitis, fibrosis, and cirrhosis [34]. To understand if gut microbiota is associated with liver fibrosis, a microbiota modification was induced by the creation of a bile duct ligation in high-fat mice models, taking into account that the bile acids have antimicrobial properties [20]. This process leads to decreased hepatic triglyceride (TG) content and increased fibrosis, thus allowing the correlation of microbiota to fibrosis and not to potential effects of fat liver content [20]. In this model, an increase in percentage of Gram-negative versus Gram-positive bacteria was also observed, a reduced ratio between Bacteroidetes and Firmicutes, as well as a dramatic increase of Gram-negative Proteobacteria. To further support the role of microbiota in liver fibrosis, high-fat-diet (HFD) microbiota was transplanted to control mice, resulting in an increase in liver injury [20].

#### 3.1.2. Clinical Data

Some studies have documented small intestinal bacterial overgrowth (SIBO) in NAFLD and NASH patients, suggesting that increased exposure to intestinal bacterial products may contribute to their pathogenesis [35, 36]. Furthermore, hepatic steatosis has been associated with increased permeability caused by disruption of intercellular tight junctions in the intestine, which was linked to SIBO [36]. Since then, growing lines of evidence have suggested that NAFLD patients are characterized by different gut microbiota composition, termed fecal dysbiosis.

To determinate the association between fecal microbiota and hepatic steatosis Wong et al. have analyzed fecal microbiota from 16 NASH patients and 22 controls [14]. NASH patients had lower fecal abundance of* Faecalibacterium* and* Anaerosporobacter*, but higher abundance of* Parabacteroides* and* Allisonella*. However, intrahepatic triglyceride content improvement was generally associated with a reduction in the abundance of Firmicutes and increase in Bacteroidetes, which reflects the contradictory data that still exists regarding the association between gut microbiota profile and hepatic steatosis.

In line, NASH children had unique characters in the composition, ecological diversity, and enterotyping patterns of gut microbiome [15]. However, when comparing NASH children, with healthy subjects and obese patients, fewer differences were observed between obese and NASH microbiomes. Among taxa with greater than 1% representation, Proteobacteria, Enterobacteriaceae, and* Escherichia* were the only phylum, family, and genus showing significant difference between obese and NASH microbiome [15].* Escherichia*, under anaerobic conditions, is capable of converting sugars to a mixture of products by fermentation, including ethanol [37]. NASH patients had elevated blood alcohol, compared to healthy subjects and obese patients [15]. Taking into account that intestinal microflora is the major source of endogenous alcohol [38], these data have supported the hypothesis that elevated representation of alcohol-producing bacteria in NASH microbiome may cause liver inflammation in NASH by a constant supply of reactive oxygen species to the liver. However, Raman et al. failed to demonstrate an increase in* Escherichia* abundance in NAFLD patients and ethanol was identified as a ubiquitous fecal volatile organic component (VOC) in both obese NAFLD adults and healthy controls; besides ester VOC were more frequently present in fecal samples from obese NAFLD patients [16]. Nevertheless, neither blood nor breath alcohol concentrations were measured and population was limited to obese NAFLD and healthy subjects, turning difficult to conclude if the observed differences in ester VOC were a consequence of NADLD rather than obesity [16].

Low-choline diets have been associated with NAFLD [24]. In this context, it was demonstrated that gut microbiota composition changes according to dietary choline levels. During choline depletion, the levels of Gammaproteobacteria and Erysipelotrichi were directly associated with liver fat content in each subject [17]. Thus, a model was created that accurately predicted the degree to which subjects developed fatty liver on a choline-deficient diet, taking into account these bacteria levels and changes in amount of liver fat and a single nucleotide polymorphism that affects choline. Taking into account that Gammaproteobacteria and Erysipelotrichi are Gram-negative bacteria, containing LPS, which was previously described as an inductor of chronic inflammation that characterize metabolic dysfunction, insulin resistance, and diabetes [39], it can therefore be hypothesized that LPS may contribute to NAFLD development in these patients.

### 3.2. Pre- and Probiotics Intervention and Their Mechanism of Action in NAFLD

#### 3.2.1. Experimental Data

Prebiotics and probiotics are known modulators of gut microbiota. The role of intestinal microbiota in NAFLD has garnered significant attention, by demonstration of the beneficial effects of pre- or probiotics administration in NAFLD models. This finding is sustained at different levels, including gut microbiota profile, gut barrier function, LPS and low-grade inflammation, lipid metabolism, and energy balance.

Supplementation of a HFD with fungal chitin-glucan (CG) decreased hepatic triglyceride accumulation and restored the number of bacteria from clostridial cluster XIVa including* Roseburia spp.,* which were decreased due to HFD [25]. CG treatment also significantly decreased HFD-induced body weight gain, fat mass development, fasting hyperglycemia, glucose intolerance, and hypercholesterolemia, independently of the caloric intake. These beneficial effects were correlated with specific bacteria of clostridial cluster XIVa, that is,* Roseburia spp*., and did not appear to be mediated by incretin glucagon-like peptide 1 (GLP-1) [25].

*Lactobacillus rhamnosus GG* (LGC) supplementation in high-fructose-induced NAFLD mice had strongly reduced liver fat accumulation [26]. The fat liver content improvement by LGC was associated with manipulation of gut microbiota: LGC have increased the total numbers of Firmicutes and Bacteroidetes; LGC attenuated the expression of the proinflammatory cytokines TNF-*α* and IL-1*β* and IL-8R in the liver. To determine whether changes in portal LPS levels and intestinal inflammation were associated with changes in intestinal barrier, the levels of occludin and claudin-1, tight junction proteins, were measured [26]. Occludin and claudin-1 expression was reduced in mice fed high-fructose diet compared to control diet and restored after LGC supplementation. These data support the hypothesis that the associated beneficial effects of increased members of the Firmicutes are due to the fact that they produce butyrate, which is known to regulate gut barrier function [40].

LPS role was further supported by other experimental studies.* Lactobacillus casei strain* (LcS) administration suppressed LPS elevation and protected against methionine-choline-diet-induced NASH development in a mice model [27]. Thus, gut modulation by LcS administration may contribute to the normalization of tight junction proteins, protects against impairment of gut permeability, and subsequently diminishes inflammation and reverse hepatic steatosis. Furthermore, treatment with a Chinese herbal formula (CHF) supplementation ameliorated NAFLD and resulted in a reduction in* Escherichia*/*Shigella* levels, Gram-negative bacteria containing LPS in their cell walls that may impair the gut barrier and trigger a low-grade chronic inflammation state [28]. CHF supplementation increased* Collinsella*, short chain fatty acid (SCFA) producers [28]. SCFA may also be responsible for the beneficial effects of CHF treatment, as SCFA can stimulate epithelial cell proliferation, which may improve gut barrier integrity [41].

Immune modulation was also observed after oral administration of* Bacteroides uniformis* CECT 7771, which reduced liver steatosis in HFD-fed mice, improved immune defense mechanisms on macrophages and dendritic cells, and reduced the gut inflammatory signals [29].

To support the effects of gut microbiota in modulation of fat storage and host metabolism, the expression of ChREBP, a transcription factor required for glucose-induced expression of the lipogenic genes acetyl-CoA carboxylase 1 (ACC1) and fatty acid synthase (FAS), as well as that of ACC2 and FAS was found to be significantly increased in mice fed with fructose-rich diet and significantly reduced after LGC [26]. Moreover, in another mice model of NAFLD, fructooligosaccharides (FOS) supplementation reduced hepatic triglyceride accumulation through changes in microbiota composition, thus leading to an increase in GLP-1, which stimulates fatty acid oxidation by peroxisome proliferator-activated receptor-alpha and lessened cholesterol accumulation by inhibiting sterol regulatory element binding proteins (SREBPs) [30].

#### 3.2.2. Clinical Data

In human, few prospective, randomized, and controlled clinical trials have yet been designed to address the potential role of intestinal microbiota in NAFLD and the potential beneficial effects from modulation of gut microbiota, by pre- or probiotics intervention.

VSL#3 is a mixture of eight probiotic strains (*Streptococcus thermophilus, bifidobacteria [B. breve, B. infantis, B. longum], Lactobacillus acidophilus, L. plantarum, L. paracasei, and L. delbrueckii subsp. bulgaricus*) [18]. In children, a 4-month supplementation with VSL#3 has improved NAFLD [18]. Therefore, it is conceivable that the effects of VSL#3 in these patients could be dependent on the restoration of normal gut microbiota. Also, circulating levels of GLP-1, both in total and in active form, have significantly increased after the 4-month supplementation, which may have improved fat metabolism. GLP-1 is an incretin secreted by L-cells in the small intestine in response to food intake, whose main roles are stimulation of glucose-dependent insulin secretion, inhibition of postprandial glucagon release, delay of gastric emptying, and induction of pancreatic *β*-cell proliferation [42]. Besides improving hepatic glucose metabolism, GLP-1 seems to be a novel target against NAFLD, by increasing fatty acid oxidation, decreasing lipogenesis, and improving hepatic glucose metabolism [42], and may also be an active intervenient, establishing the link between NAFLD and gut microbiota.

Treatment with* Bifidobacterium longum* plus fructooligosaccharides (Fos) reduced HOMA-IR and NASH activity in association with reduced endotoxin, C-reactive protein, and TNF-*α* levels [19]. These data further support the hypothesis that endotoxin-induced activation of macrophages plays a key role in the pathogenesis of liver injury in NAFLD patients.

## 4. Discussion

NAFLD is an emerging complex multifactorial disease resulting from the interaction of genetic, environmental, metabolic, and inflammatory factors. Both obesity and diabetes are major risk factors for NAFLD [43]. As it has been previously described, the gut microbiota exerts a profound influence on fat deposition, being a key regulator of energy storage [11, 44].

Germ-free mice colonized with gut microbiota from obese animals showed body fat mass and liver triglyceride content and an insulin resistance increase. Microbiota promoted absorption of monosaccharides from the gut lumen, with resulting induction of* de novo* hepatic lipogenesis, by increased activity of acetyl-CoA carboxylase and fatty acid synthase [11]. In humans, this relationship is further reinforced by the demonstration of the relative fewer proportion of Bacteroidetes in obese people by comparison with lean people and the shift toward higher relative abundance of Bacteroidetes and decreased number of Firmicutes in obese patients losing weight through low-calorie diets [9]. These named “obese microbiomes” have increased capacity of harvesting energy from food, resulting in fat accumulation. However, the relationship between Bacteroidetes and Firmicutes levels and obesity and associated metabolic disturbances is still controversial.

According to what has been described in Section 3, differences in gut microbiota profile may also have impact on the liver, on the background of obesity and insulin resistance. Most of the available data demonstrating this association is based on association studies, lacking human intervention studies, which would further improve the knowledge of gut microbiota influence on NAFLD.

As it has been described, insulin resistance is a common feature of metabolic syndrome and NAFLD. Thus, the decrease of the inhibitory effects of insulin on peripheral lipolysis increases the availability of free fatty acids, playing a critical role in the development of fatty liver [45]. Metabolic endotoxemia triggers insulin resistance, obesity, and diabetes, through LPS, which in combination with CD14 serves as ligand for TLR [46]. LPS and other endotoxins also can activate TLRs, inducing an inflammatory response, linked to hepatic fat accumulation [20, 21, 31, 32]. An interesting finding was the observation that small intestinal bacterial overgrowth predicted severe hepatic steatosis [47]. In fact, bacterial overgrowth may increase intestinal permeability, by disruption of intercellular tight junctions, subsequently exposing liver surface to bacterial products, resulting in hepatic fat deposition [36].

An additional contributor is the modulation of choline metabolism by intestinal microbiota. Choline and methionine-deficient diets have been associated with hepatic steatosis [17, 48]. The gut microbiota catalyzes the conversion of choline to dimethylamine and trimethylamine [49]. A high-fat diet in a mice model susceptible to impaired glucose homeostasis and NAFLD reduces the bioavailability of choline, mimicking the effect of choline-deficient diets [24]. These results establish a possible association between choline bioavailability and hepatic steatosis, through metabolic activity of gut microbiota, which is affected by diet. In addition, Gammaproteobacteria and Erysipelotrichi levels were associated with hepatic steatosis, during choline depletion [17]. As these bacteria are Gram-negative, these data further support the role of LPS as an active player on NAFLD development.

Endogenous production of ethanol by bacteria also seems to mediate hepatic fat accumulation. In an obese mouse model, in the absence of ethanol ingestion, ethanol was detected in exhaled breath [50]. Hence, intestinal production of ethanol may contribute to the genesis of obesity-related fatty liver, triggering inflammatory signals [15].

Interventional studies with pre- and probiotics gave further support to the possible effects of intestinal microbiota modulation on NAFLD pathogenesis. Besides the impact on fat storage and host metabolism, GLP-1 may be an important contributor, linking NAFLD, insulin resistance, and gut microbiota. FOS supplementation in a mice model of hepatic steatosis reduced fatty liver accumulation, through changes in gut microbiota, responsible for GLP-1 increase [30]. GLP-1 stimulated fatty acid oxidation by peroxisome proliferator-activated receptor-alpha and inhibited SREBPs [30]. In children, VSL#3 supplementation improved NAFLD and had increased GLP-1, supporting the impact of gut microbiota modulation on fat metabolism [18].

**Figure 3:**
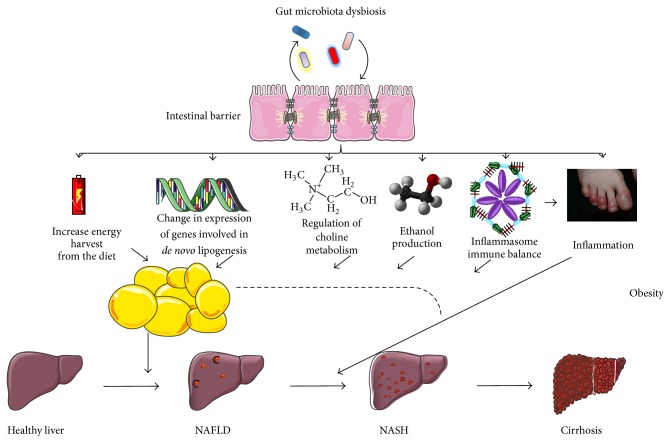
Possible pathways involved in NAFLD pathogenesis, by gut microbiota.

Therefore, intestinal microbiota, beyond its capacity to regulate body fat gain and insulin resistance, seems to play a fundamental role on NAFLD, through different pathways (Figure 3), including

- increasing energy harvest from diet,
- change in expression of genes involved in* de novo* lipogenesis,
- regulation of choline metabolism,
- ethanol production,
- inflammasome and innate immunity,
- inflammation.

However, the majority of studies were conducted under experimental conditions, namely, under fat rich diets, which limits the demonstration of a definitive role of gut microbiota in hepatic steatosis, especially in NAFLD nonobese patients. Further comprehension of the relationship between gut microbiota and hepatic steatosis will allow the development of new specific targets and integrated strategies to modulate intestinal microbiota, including prebiotics and probiotics, in order to improve or even cure this prevalent metabolic disease.

## Figures and Tables

**Table 1:** Clinical studies on NAFLD and gut microbiota in humans.

Study patients and methodology	Outcomes	Reference number
Randomized controlled trial of 38 patients, 16 NASH patients (7 supplemented with probiotic versus 9 usual care group) versus 22 controls	NASH patients had lower fecal abundance of *Faecalibacterium* and *Anaerosporobacte*r but higher abundance of *Parabacteroides and Allisonella *	[14]

Cross-sectional study of 63 children, 16 controls versus 25 obese versus 22 NASH patients	Proteobacteria/Enterobacteriaceae/*Escherichia* was similarly represented between healthy and obese microbiomes but was significantly elevated in NASH	[15]

Cross-sectional study of 60 patients, 30 NAFLD patients versus 30 controls	Lactobacillus and selected members of phylum Firmicutes (*Dorea*, *Robinsoniella*, and *Roseburia*) were higher in NAFLD patients; Oscillibacter was underrepresented	[16]

In-patient study of 15 female subjects placed on well-controlled diets in which choline levels were manipulated	Variations between levels of Gammaproteobacteria and Erysipelotrichiwere directly associated with changes in liver fat in each subject during choline depletion	[17]

Randomized controlled trial of 48 children with NAFLD-22 supplemented with VSL#3 versus 22 placebos	A 4-month supplementation with VSL#3 improved NAFLD in children	[18]

Randomized controlled trial of 66 patients with NAFLD-34 supplemented with *Bifidobacterium longum* with fructooligosaccharides (FOS) and lifestyle modification versus 32 lifestyle modifications alone	*Bifidobacterium longum* with FOS and lifestyle modification significantly reduces endotoxin, hepatic steatosis, and NASH activity index	[19]

**Table 2:** Experimental studies on NAFLD and gut microbiota in mice.

Model	Outcome	Reference no.
High-fat diet- (HFD-) fed mice versus controls subjected to bile duct ligation (BDL) or hepatotoxin CCl4	HFD mice subjected to BDL had an increase of Bacteroidetes, Firmicutes, and Proteobacteria	[20]

Methionine-choline-deficient diet-fed mice versus HFD-fed mice	Inflammasome deficiency-associated changes in gut microbiota were associated with exacerbated hepatic steatosis and inflammation	[21]

HFD-fed germ-free mice colonized with intestinal microbiota from a responder donor (developed hyperglycaemia and higher proinflammatory cytokines) or a nonresponder	Responder-receiver developed hepatic macrovesicular steatosis and harbour distinct gut microbiota	[22]

Low-fat diet based on palm oil (LFD-PO) fed mice versus HFD based on palm oil (HFD-PO) versus olive oil (HFD-OO) versus safflower oil (HFD-SO)	The HFD-PO diet induced higher liver triglyceride content, reduced microbial diversity, and increased the Firmicutes-to-Bacteroidetes ratio	[23]

HFD-fed mice versus low-fat diet-fed mice	Quantitative variation in dietary choline induced an inverse quantitative variation in liver fat content; conversion of choline into methylamines by microbiota in mice on a HFD caused NAFLD	[24]

HFD-fed mice versus HFD supplemented with chitin-glucan (CG) versus controls	CG treatment significantly decreased hepatic triglyceride accumulation, which was negatively correlated with specific bacteria of clostridial cluster XIVa, that is, *Roseburia spp. *	[25]

High-fructose diet-fed mice supplemented with *Lactobacillus rhamnosus GG* (LGC) versus controls	Supplementation with LGC reduced liver fat accumulation and increased intestinal Firmicutes and Bacteroidetes	[26]

Methionine-choline-deficient-diet-fed mice (MCD) versus MCD-fed mice supplemented with *Lactobacillus casei* strain Shirota (LcS) versus controls	*Bifidobacterium* and *Lactobacillus *were markedly reduced by the MCD diet. Administration of LcS increased the *L. casei* subgroup and other lactic acid bacteria	[27]

HFD-fed rats supplementation with an herbal formula (HF) versus no supplementation versus controls	Supplementation of HF decreased hepatic steatosis; *Escherichia*/*Shigella* were enriched in HFD-fed rats but decreased to control levels after HF treatment	[28]

HFD-fed mice supplemented with *Bacteroides uniformis* CECT7771 versus controls	Supplementation with *Bacteroides uniformis* reduced NAFLD in HFD-mice; HFD resulted in marked changes in gut microbiota, partially restored by the intervention	[29]

N-3 PUFA-depleted diet-fed mice supplemented with fructooligosaccharides (FOS) versus controls	Supplementation with FOS reverses NAFLD induced by n-3 PUFA-depleted diet; FOS-treated mice exhibited higher caecal *Bifidobacterium spp*. and lower *Roseburia spp.* content and reduced hepatic triglyceride accumulation	[30]

Fructose-fed mice versus controls treated or not with antibiotics	Hepatic fat accumulation was associated with a significant induction of TLR 1–4 and 6–8. The effects of fructose were attenuated in antibiotic-treated mice. No systematic alterations of microbiota were found	[31]
